# Phosphate Activation via Reduced Oxidation State Phosphorus (P). Mild Routes to Condensed-P Energy Currency Molecules

**DOI:** 10.3390/life3030386

**Published:** 2013-07-19

**Authors:** Terence P. Kee, David E. Bryant, Barry Herschy, Katie E. R. Marriott, Nichola E. Cosgrove, Matthew A. Pasek, Zachary D. Atlas, Claire R. Cousins

**Affiliations:** 1 School of Chemistry, University of Leeds, Woodhouse Lane, Leeds, LS2 9JT, UK; E-Mails: d.e.bryant@leeds.ac.uk (D.E.B.); b.herschy@ucl.ac.uk (B.H.); katie.marriott@glasgow.ac.uk (K.E.R.M.); n.e.cosgrove@leeds.ac.uk (N.E.C.); 2 Department of Geology, University of South Florida, 4202 East Fowler Ave., SCA 528, Tampa, FL 33620, USA; E-Mails: mpasek@usf.edu (M.A.P.); zatlas@usf.edu (Z.D.A.); 3 Department of Earth and Planetary Science, Birkbeck College, University of London, Gower Street, London, WC1E 6BT, UK; E-Mail: c.cousins@ucl.ac.uk; 4 UK Centre for Astrobiology, School of Physics and Astronomy, University of Edinburgh, Edinburgh, EH9 3JZ, UK

**Keywords:** phosphorus, prebiotic chemistry, origin of life, meteorites

## Abstract

The emergence of mechanisms for phosphorylating organic and inorganic molecules is a key step *en route* to the earliest living systems. At the heart of all contemporary biochemical systems reside reactive phosphorus (P) molecules (such as adenosine triphosphate, ATP) as energy currency molecules to drive endergonic metabolic processes and it has been proposed that a predecessor of such molecules could have been pyrophosphate [P_2_O_7_^4−^; PPi(V)]. Arguably the most geologically plausible route to PPi(V) is dehydration of orthophosphate, Pi(V), normally a highly endergonic process in the absence of mechanisms for activating Pi(V). One possible solution to this problem recognizes the presence of reactive-P containing mineral phases, such as schreibersite [(Fe,Ni)_3_P] within meteorites whose abundance on the early Earth would likely have been significant during a putative Hadean-Archean heavy bombardment. Here, we propose that the reduced oxidation state P-oxyacid, H-phosphite [HPO_3_^2−^; Pi(III)] could have activated Pi(V) towards condensation via the intermediacy of the condensed oxyacid pyrophosphite [H_2_P_2_O_5_^2−^; PPi(III)]. We provide geologically plausible provenance for PPi(III) along with evidence of its ability to activate Pi(V) towards PPi(V) formation under mild conditions (80 °C) in water.

## 1. Introduction

Amongst the most important and ubiquitous energy-currency molecules of contemporary biochemistry are activated phosphorus (P) species such as phosphocreatine, phosphoenol pyruvate, and adenosine triphosphate (ATP) [1]. These molecules are able to selectively discharge tranches of energy (*ca*. 40 kJmol^−1^ for the hydrolysis of ATP to ADP) when coupled, mechanistically, to drive endergonic chemical reactions [1], the supply of ATP being recharged via mitochondrial oxidative phosphorylation [2] and substrate-level phosphorylation during glycolysis [3]. A significant problem in the field of abiogenesis concerns the emergence of a global P-based system of bioenergetics based on condensed phosphate energy currency molecules such as ATP. In addition to its role in RNA synthesis, so firmly embedded is ATP in cellular bioenergetics that it is not unreasonable to envisage P-based bioenergetics being amongst the most ancient of biochemical machinery [4]. Some challenging problems emerge: (i) could simpler P-based systems have preceded ATP as energy currencies, (ii) how could such systems have emerged within early earth geological environments, (iii) what chemical processes could such energy currency molecules have driven, and (iv) how might such primitive P-based systems have evolved chemically into ATP-based contemporary biochemistry? Pyrophosphate [PPi(V); P_2_O_7_^4−^] has been proposed as a logical ancestor of ATP [4,5,6], not in the least because of a firmly established role for the former in biology [7,8]. Not only does PPi(V) retain the key [P-O-P] linkage of ATP, central to energy transduction, but divested of its adenosine cloak, the prebiotic plausibility of PPi(V) becomes associated less with RNA world chemistry and more focused on terrestrial geology. Pyrophosphate can be considered an example of an energy currency molecule of relatively high kinetic stability [9]. We presume that PPi(V), and by association polyphosphates [10], would have emerged as key, relatively kinetically stable [11], energy currencies only when catalyst systems were available [12] to use them effectively in P-transfer chemistry and energy release. The search for geologically plausible, low energy routes to PPi(V) has led to some exciting developments including mild formation mechanisms (160–180 °C) within hydrothermal vent-like conditions [13] and a recognition that other, more chemically reactive condensed phosphorus compounds may have both (i) preceded PPi(V) and (ii) permitted a low-energy chemical pathway directly to PPi(V). Cyclotrimetaphosphate (cTMP; P_3_O_9_^3−^) is one such example; a prebiotically plausible, energy currency molecule with a rich chemistry [14] and lower kinetic stability than PPi(V), it undergoes hydrolysis to PPi(V). Its presence is implied within volcanic environments [15] and it has the significant advantage of facilitating chemical reactions without the need for complex catalysis, including peptide synthesis [1] and phosphorylation of organics [16], although its geochemical provenance has been questioned [10]. Here, we introduce a method of activating Pi(V) based on the reduced oxidation form of P, H-phosphite, Pi(III). As part of this mechanism, the condensed oxyacids pyrophosphite [PPi(III); H_2_P_2_O_5_^2−^] and isohypophosphate [PPi(III–V); H_2_P_2_O_6_^3−^] are seen as playing significant roles. In this contribution we provide geologically plausible provenance for both Pi(III) and PPi(III) along with evidence of the ability of the latter to provide a low energy route to PPi(V) via activation of Pi(V) towards condensation in the presence of divalent metals.

## 2. Experimental Section

### 2.1. Materials and General Analytical Methods

Water was purified by ion exchange on a Purite Select Analyst (PSA) reverse osmosis-deionization system (Purite Ltd., Oxford, UK). D_2_O for NMR analyses was used as received from Sigma-Aldrich. Solutions of aqueous HCl, NaOH and Na_2_S were prepared by dilution of commercial samples in PSA deionized water. Solution pH measurements were made on a Schochem pH meter buffered to pH 4 and 7 with commercial (Fisher Chemicals) standards. ^31^P-NMR analyses were performed on a Bruker Avance 500 MHz instrument operating at 202.634 MHz for ^31^P internally referenced to 85% H_3_PO_4_. Iron, principally in the form of ferrous (Fe^2+^) for our Icelandic field samples, was removed from all samples prior to NMR analysis to alleviate the problems associated with paramagnetic broadening. This was done by pH adjustment, first to *ca*. 12 by addition of NaOH (aq) (1 M) which leads to precipitation of oxides of iron, followed by addition of aqueous Na_2_S solution (1 M), centrifugation, filtration and re-adjustment back to pH *ca*. 4 with HCl (1 M). For each sample, 10 mL of fluid were reduced to dryness and the residue redissolved in 0.5 mL deionized water or D_2_O, filtered using 0.45 μm syringe filters and analyzed. For those samples run in H_2_O solvent, D_2_O inserts were used to provide a deuterium lock. Samples within which pyrophosphite, PPi(III), was expected to be present and analyzed were pH adjusted to between 7–8 by addition of NaOH_aq_ (1 M).

### 2.2. Production of Pyrophosphite PPi(III)

Solutions of H_3_PO_3_ (50 mM) in deionized water were pH adjusted to 4.5, 5.0, 5.5, and 5.8 with aqueous NaOH (50 mM) solution before being evaporated to dryness, dried for several h in a Belling open oven at 50 °C, ground to a fine powder and then placed in a vacuum oven set to 85 °C, 1–10 mbar pressure for 4, 5, and 6 days. After this time, an aliquot (*ca*. 50 mg) was removed, dissolved in distilled water, pH adjusted by addition of NaOH (1 M) solution to between 7–8 to mitigate against acid-catalyzed hydrolysis of PPi(III) and analyzed, in triplicate, by ^31^P-NMR spectroscopy (data are collected in Table 1). Solutions of H_3_PO_3_ (0.1 M) in standard mean ocean water (SMOW; minus Fe^2+^ which compromises NMR analysis) were pH adjusted to 3.0 and 4.0 with aqueous NaOH (0.1 M) solution before being evaporated to dryness, dried for several h in a Belling open oven at 50 °C, and then placed in a vacuum oven set to 85 °C, 1–10 mbar pressure for several days (up to 2 weeks). After this time, an aliquot (*ca.* 50 mg) was removed, dissolved in distilled water, pH adjusted by addition of NaOH (1 M) solution to between 7–8 to mitigate against acid-catalyzed hydrolysis of PPi(III) and analyzed by ^31^P-NMR spectroscopy in triplicate (Table 1).

**Table 1:** (*upper*) Conversion (%) of Pi(III) (50 mM) to PPi(III) via evaporation and low pressure heating (85 °C, 1–10 mbar) from deionized water as a function of starting pH; (*lower*) Conversion (%) of Pi(III) (100 mM) to PPi(III) via evaporation and low pressure heating (85 °C, 1–10 mbar) from standard mean ocean water as a function of time (samples analyzed in triplicate, with standard deviations in parentheses).

pH	4 Days	5 Days	6 Days
4.0	24(2)	24(4)	30(1)
4.5	12(0)	14(1)	15(1)
5.0	8(1)	10(1)	17(0)
5.5	12(0)	14(1)	15(1)
5.8	4(1)	5(0)	6(0)
6.0	0	0	0
**Time (h)**		**pH 3**	**pH 4**
24		61(4)	39(3)
48		61(3)	16(1)
72		62(4)	20(10)
336		35(5)	38(0)

### 2.3. Hveradalur Lake Geothermal Field Experiments: Site

Beneath the northern margin of the Vatnajökull glacier in Central Iceland lies the Kverkfjöll volcanic system [17]. There are significant geothermal areas surrounding the rim of the northern caldera, some of which have been described previously [18], which display a range of temperatures and pH’s. Of most significance to our investigations here were the low pH (1–5) geothermal fluids of the Hveradalur geothermal area (64°40.173'N; 16°41.100'W) sampled during a June 2011 field expedition. The Kverkfjöll volcano system consists of two sub-glacial volcanic calderas, elliptical in shape and approximately 8 km × 5 km in size. The main area of interest is situated on the western edge of the northern caldera, which contains a 3 km long by 1 km wide geothermal zone. Though much of this area is inaccessible due to steep and unstable cliffs, there are partially accessible regions which contain glacial melt lakes and hydrothermal and geothermally active areas which made this site of particular interest due to the wide and varied conditions reported of, for example, temperature, pH and redox potential. A full description of this site, associated geology, water chemistry and similarities to Martian environments has recently appeared [19].

### 2.4. Hveradalur Lake Geothermal Field Experiments: ICP-AES and ICP-MS-HPLC Analyses

Eight samples of Fe_3_P were prepared in 50 mL capacity Falcon tubes and treated with various Hveradalur lake geothermally heated fluids (50 mL) for various time periods under different pH and temperature conditions. Samples A1, 3, 5, 6, 7, 8 were incubated with initially hot fluids for four days but were allowed to cool to ambient temperature and left for between 82–86 days prior to analysis (non-isothermal conditions). Samples A2 and 4 were incubated in the fluids noted at the natural temperatures for 4 days (A2) and 2 days (A4) respectively prior to work up for analysis via ICP-AES or ICP-MS methods through a sequence of steps as outlined below:

(i) Samples were pre-filtered using Whatman Number 1 filter papers.
(ii) Samples were then diluted to 70 mL and re-filtered using Pall Corporation Acrodisc 32 mm 0.45 µm syringe filters to remove un-dissolved particulates.
(iii) Nalgene bottles (30 mL capacity) were acid washed by filling with 50% HCl and leaving for 24 h. They were rinsed 3 times with tap water and 3 times with deionized water then left to air dry.
(iv) Two portions of 35 mL of sample were placed in an acid-washed 30 mL Nalgene bottle.
(v) Concentrated nitric acid (*ca*. 2 drops) was added to one of the portions and the lids screwed on tightly and the bottles labeled.
(vi) Due to the concentration of sulphur, the acid treated samples were further diluted to allow accurate measurement of the sulphur content of each sample.
(vii) Two 1.5 mL aliquots were taken from each of the acid treated samples. Two drops of toluene was added as an antibacterial agent. The sub-samples were subsequently submitted for ICP analysis.

Fluids from a variety of sampling sites inthe Hveradalur geothermal area were pre-filtered to remove suspended particulate matter followed by fine-filtration using a Millipore 0.45 μm filter (post-collection in the UK). Duplicate 30 mL water samples were taken, one of which was acidified with nitric acid, and these were analyzed with a Dionex Ion Chromatograph and Horoba JY Ultima 2C ICP-AES for dissolved anion and cations respectively, at the Wolfson Geochemistry Laboratory at Birkbeck College—UCL. Cation results were taken as the mean of three repeat measurements, with standard deviations typically between 0.01–0.1 mg L^−1^. Three standards were run for P analysis and sample standard deviations included accordingly. Data are collected in Table 2. Methods for ICP-MS and for P speciation by HPLC-ICP-MS analysis at USF were relatively straightforward. Sample aliquots were diluted 1:10 in analytical grade I water (18 MΩ) and run into a Perkin-Elmer Elan DRC II ICP-MS with an in-line addition of indium used as an internal standard. Samples were calibrated against a set of synthetic P standards made from a serial dilution of a 1,000 mg/mL aqueous standard (High Purity Standards). The ICP-MS was run at higher RF power (1,300 W) to more effectively ionize phosphorus, which has a high first ionization potential. Nebulizer flow and lens voltage was adjusted to optimize for maximal phosphorus counts. Data are collected and compared to ICP-AES values in Table 2, again with SSD’s from triplicate determinations. Speciation was performed on a Perkin-Elmer S200 HPLC using a Dionex Ion Pac^®^ AS17C chromatographic column preceeded by an AG17 Guard Column. This method represents a new application for ICP-MS and will be discussed in detail in a forthcoming paper. General methods were modified from those used for P speciation on IC-Electro spray MS and optimized for HPLC-ICP-MS. Sample aliquots (50 μL) were eluted with KOH using a linear concentration gradient at 1.5 mL/min flow rate. P was detected on the ICP-MS as mass 31 and the ICP-MS was optimized as before. Calibration standards were made from reagent grade or better synthetic H-phosphorous [identified as P(III)] and orthophosphoric [identified as P(V)] acids and were mixed just prior to analysis. Calibrations were performed on orthophosphate, H-phosphite and hypophosphite at 0.3, 3.0 and 30 μM and calibration curves yielded r^2^ better than 0.995.

**Table 2:** ICP-AES and ICP-MS analyses of elemental phosphorus in aqueous samples resulting from Fe_3_P samples (A1–8) incubated in Hveradalur Lake geothermal fluids. **^ζ^** In parentheses (site location; location temperature in °C; location pH); **^†^** ESD’s in parentheses calculated from triplicate runs; **^‡^** Sample standard deviations in parentheses calculated from triplicate-runs.

Sample ^ζ^	ICP-AES ^†^ (μgL^−1^)	ICP-MS ^‡^ (μgL^−1^)
Blank 1	–0.04(1)	–0.02(0)
A1 (KHL–UCL3; 40; 3.6)	0.53(2)	0.39(3)
A2 (KHL–BPR; 79.5; 4.0)	1.01(3)	0.92(0)
A3 (KHL–MP1; 87.4; 1.6)	21.98(3)	17.17(5)
A4 (KHL–LP1; 93.5; 3.1)	2.66(2)	1.03(5)
A5 (KHL–UCL5; 89.2; 4.7)	1.20(2)	1.50(4)
A6 (KHL–LP3; 79.2; 2.5)	0.62(0)	0.47(1)
A7 (KHL–LP4; 87.8; 3.3)	0.77(2)	0.70(2)
A8 (KHL–MP3; 84.7; 2.7)	2.28(6)	2.00(3)

### 2.5. Hveradalur Lake Geothermal Field Experiments: ^31^P-NMR Analyses

All Iceland samples for ^31^P-NMR analysis were prepared as follows. Pre-heated samples were analyzed after steps (i)–(iv) and post-heated subsequently after steps (vi)–(ix).

(i) A 10 mL aliquot of the acidified sample (prepared as above) was taken in a 15 mL Falcon tube.
(ii) Sample was treated with NaOH (1 M) to pH 12 and left for 1–2 min.
(iii) Sample was gravity filtered to remove precipitate (hydrated ferrous oxides and hydroxides). These oxides were subsequently collected and shown to contain negligible amounts of phosphorus via EDX measurements.
(iv) Sample was treated with HCl (1 M) to pH 4.
(v) A 0.5 mL aliquot was taken and analyzed by ^31^P-NMR spectroscopy (500 MHz Bruker Avance, 320 scans, 300 K) using capillary D_2_O inserts.
(vi) Sample was reduced to dryness and residues dried overnight in 50 °C oven.
(vii) Residues were ground to fine powder in mortar and pestle.
(viii) Residues were dry heated to *ca.* 90 °C for 72 h on a sand bath under flowing N_2_ (*ca.* 1 bubble per second).
(ix) Residues were dissolved in deionized water (*ca.* 0.5 mL) and adjusted to pH 7.2 using aqueous Na_2_CO_3_ solution (1 M).
(x) Sample was analyzed by ^31^P-NMR spectroscopy (500 MHz Bruker Avance, 2048 scans, 300 K) using capillary D_2_O inserts.

All of the pre-heated samples, (Table 2) reveal the clear presence of Pi(III) and in some cases Pi(V) as indicated also in the HPLC analyses (Section 2.4). However, the post-heated A-samples are more varied in their ^31^P-NMR responses, we presume due to the presence of trace amounts of iron in the system. Nevertheless, heated samples A1 and A5 show clear PPi(III) present, confirmed by addition of authentic samples and there is evidence for PPi(III) presence in the remaining three A samples 2, 3, and 4. Samples A6–8 did not show any PPi(III) after the above heating protocol but we do have evidence that there are several factors that affect PPi(III) production in the solid phase such as degree of dryness and particle size of the heated material. These and other variables in the solid phase dehydration of Pi(III) to PPi(III) are currently under investigation.

### 2.6. Conversion of PPi(III) to PPi(III–V)

**Figure 1:**
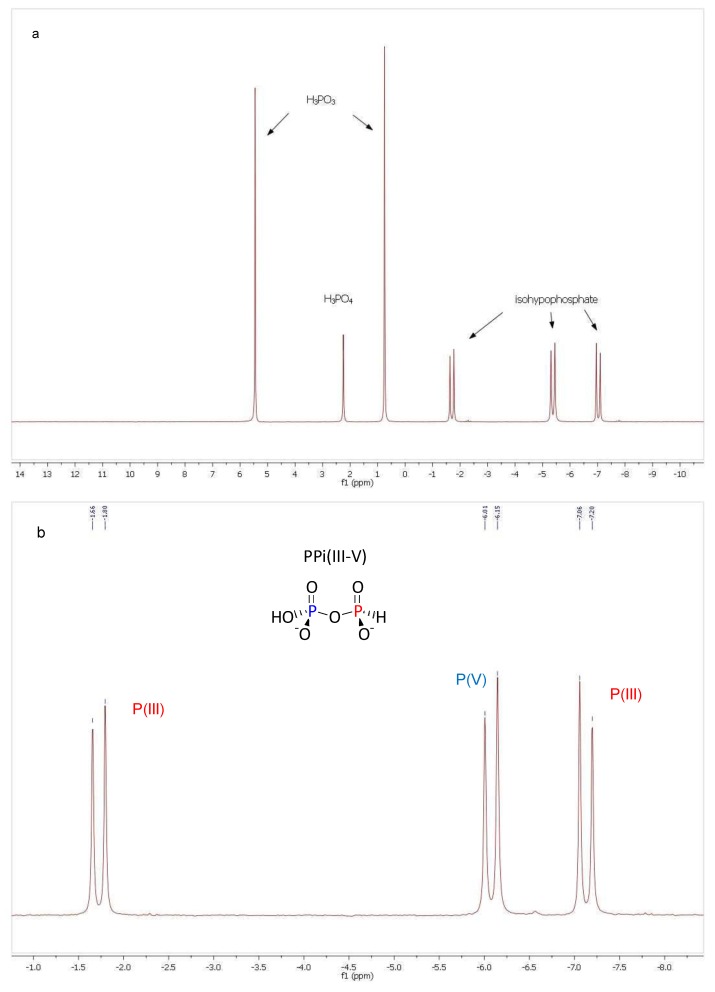
(**a**) ^31^P-NMR (298 K, H_2_O solvent with D_2_O insert, 320 scans) of a sample of PPi(III) (15 mmol) and Pi(V) (17 mmol) after 48 h incubation, 15 °C at pH 7 showing clearly isohypophosphate formation; (**b**) ^31^P-NMR of PPi(III–V) (298 K, H_2_O; pH 8; 320 scans): δ–4.43 [dd, ^1^J_PH_ = 653 Hz, ^2^J_PP_ = 17 Hz, Pi(III)]; –6.08 [d, ^2^J_PP_ = 17 Hz, Pi(V)].

Sodium pyrophosphite, Na_2_-PPi(III), reacts readily with aqueous solutions of sodium phosphate to give sodium isohypophosphate, Na_2_-PPi(III–V) (Figure 1). The reaction is neither particularly sensitive to pH nor temperature but the hydrolysis of PPi(III) to Pi(III) is always competitive, resulting in mixtures of Pi(III), Pi(V), and PPi(III–V). Yields of PPi(III–V) are therefore maximized by adjusting the pH to 7, using concentrated solutions and lowering the temperature, all of which slows this competitive hydrolysis. A typical reaction proceeds as follows: Disodium hydrogen phosphate Na_2_-Pi(V) (3.0 g, 0.017 mol) is dissolved with gentle warming in 10 mL deionized water and allowed to cool to room temperature. To this is added Na_2_-PPi(III) (2.85 g, 0.015 mol) and the flask stoppered and allowed to stand in a cool place at temperatures in the range 5–15 °C for 48 h. The PPi(III) does not immediately dissolve. After this time the phosphorus speciation is measured by ^31^P-NMR spectroscopy. Normally, a few percent PPi(III) remain which can be allowed to hydrolyze by raising the pH to 8 leaving a solution of approximately 46% of total phosphorus being present as PPi(III–V) with the remainder as Pi(III) and Pi(V). In order to reduce the competitive hydrolysis pathway of PPi(III) still further and raise the yield of PPi(III–V), a large excess of Pi(V) can be used as described by Blaser and Worms [20]. In a similar reaction to that described above the rate of phosphonylation was measured as a function of time and compared to the rate with added magnesium (as MgCl_2_). Thus, disodium hydrogen phosphate (2.50 g, 0.015 mol) is dissolved with gentle warming in 10 mL deionized water and allowed to cool to room temperature. To this, sodium pyrophosphite, Na_2_-PPi(III) (2.85 g, 0.015 mol) is added and the mixture is divided in two equal aliquots. To one aliquot was added MgCl_2_·6H_2_O, (0.5 g, 0.0025 mol) and the speciation by ^31^P-NMR was measured at time intervals. The rate of conversion is increased by the presence of magnesium but the overall yield is limited by the availability of Pi(V). Additionally, the hydrolysis of PPi(III–V) appears to be facilitated by the presence of magnesium in line with separate experiments into the hydrolysis of PPi(III–V)The above represents data from a molar ratio [PPi(III)]:Mg of *ca*. 6:1. If more magnesium is added, then PPi(III–V) formation is accelerated (*vide infra*).

### 2.7. Conversion of PPi(III–V) to PPi(V)

**Figure 2:**
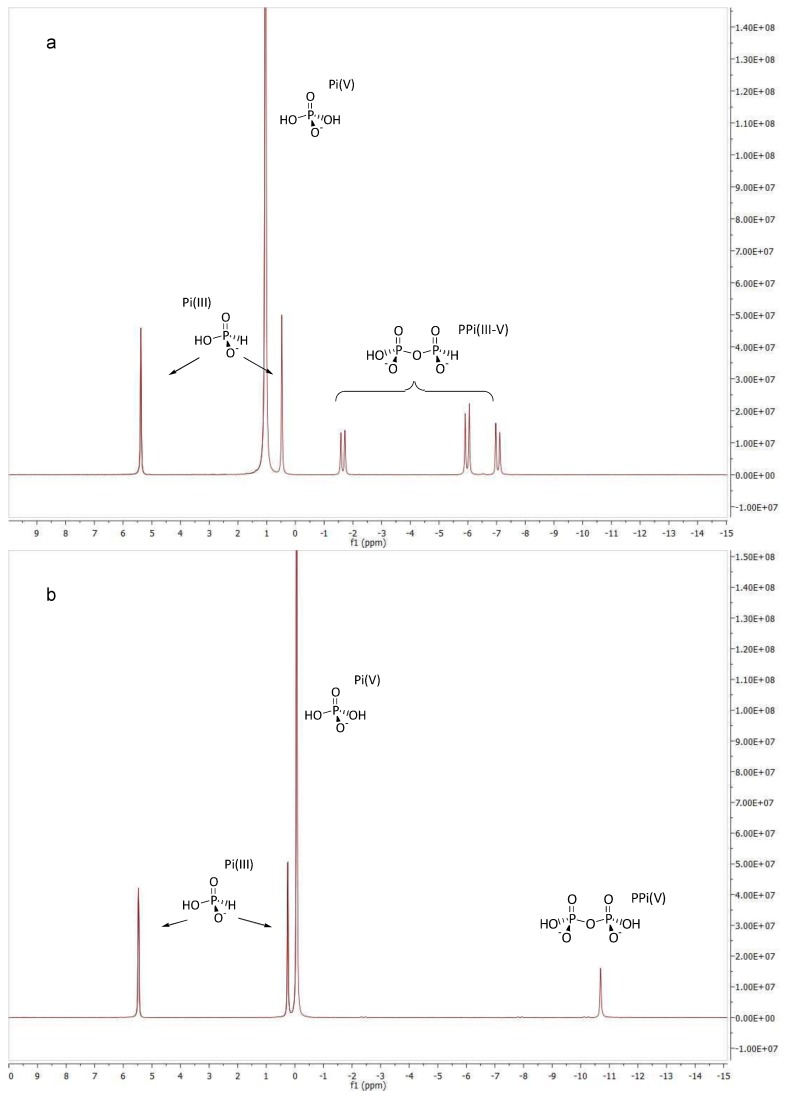
^31^P-NMR spectra (298 K, H_2_O solvent with D_2_O insert, 320 scans) of: (**a**) a starting sample with the P-speciation PPi(III–V) 43.5%, Pi(V) 6.9% and Pi(III) 49.5% (0.1 M in total P); (**b**) Sample (a) after heating in solution to 80 °C for 42 h.

PPi(III–V) reacts further with Pi(V) to give PPi(V) but under more forcing conditions (*vide infra*) and in lower overall yields. In the absence of magnesium no reaction appears to take place in solution. A solution containing PPi(III–V) 43.5%, Pi(V) 6.9% and Pi(III) 49.5% (1.5 M in total phosphorus) was diluted 6-fold to give a stock solution approximately 0.1 M in PPi(III–V). To this was added disodium hydrogen phosphate (2.04 g, 0.012 mol) and MgCl_2_·6H_2_O (3.6 g, 0.018 mol) thereby making a solution with two equivalents of Pi(V) and three equivalents of Mg^2+^ (Figure 2a). The solution was heated in a stoppered flask for 42 h at 80 °C. After *ca*. 2 h, a precipitate appeared and at the end of the experiment the flask was allowed to cool to room temperature and the pH was adjusted to 3 (1 M aqueous HCl) with stirring. The precipitates slowly dissolved and the phosphorus speciation was determined using ^31^P-NMR spectroscopy. The solution contained PPi(V) as 1.7% total phosphorus irrespective of the starting pH being 6 or 7 and all PPi(III–V) had been consumed. Approximately 7% of the PPi(III–V) has been phosphorylated to PPi(V) (Figure 2b). In control experiments, it was found that adjustment of PPi(V) solutions to pH 3 did not result in hydrolysis over a 24 h period, thus confirming the degree of acid-stability of product PPi(V) under these conditions. 

## 3. Results and Discussion

### 3.1. Pyrophosphite Formation and Geological Provenance

**Figure 3:**
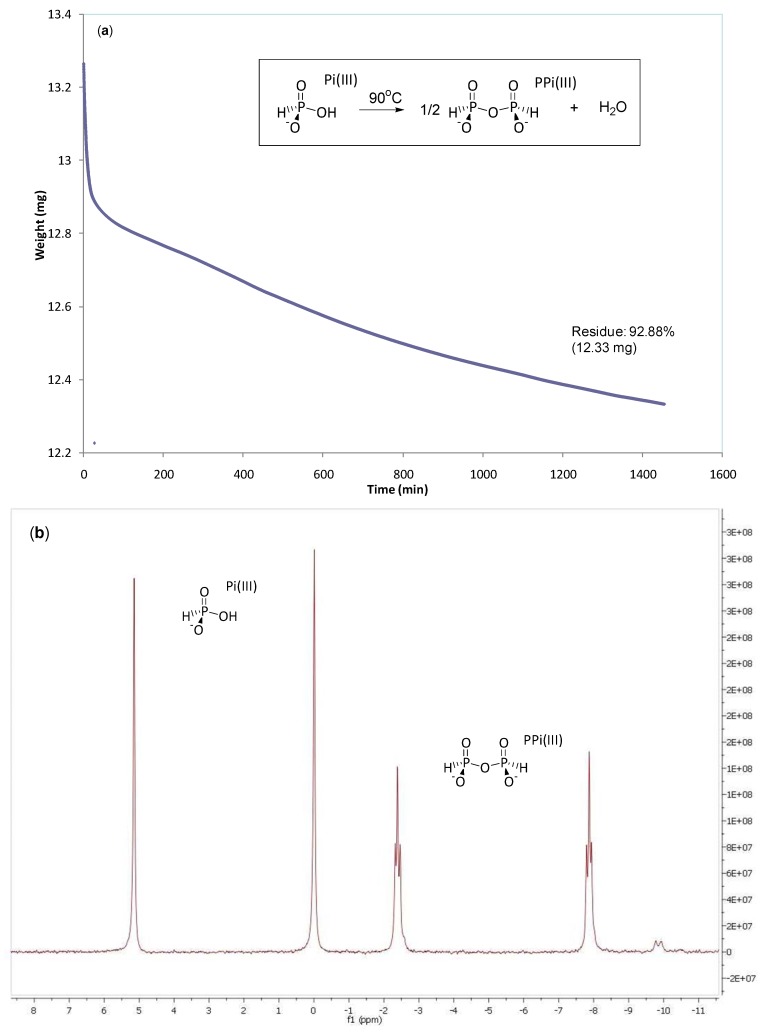
(**a**) Isothermal TGA for NaH_2_PO_3_ Pi(III) (90 °C; N_2_ gas flow 60 mL min^−1^) showing dehydration to Na_2_H_2_P_2_O_5_ Pi(III). (**b**) ^31^P-NMR (H_2_O; pH *ca*. 4) for product from TGA showing *ca*. 53:47 Pi(III):PPi(III) ratio.

Pyrophosphate [PPi(V)] can be formed by dry heating orthophosphate salts [NaH_2_PO_4_ or CaHPO_4_; Pi(V)] between 250–600 °C [21]. However, substituting orthophosphate with phosphite [or H-phosphonate, H_2_PO_3_^−^; Pi(III)] salts allows dehydration to PPi(III) under far milder conditions. Thus, isothermal thermogravimetric analysis (TGA; Figure 3a) of NaH_2_PO_3_ at 90 °C for 24 h affords *ca*. 7.1% weight loss which correlates to *ca*. 82% conversion to Na_2_-PPi(III). ^31^P-NMR analysis of a non-pH adjusted sample of this material confirms PPi(III) formation with a characteristic AA’XX’ spin system (Figure 3b) [22]. Formation of PPi(III) from Pi(III) is strongly dependent upon pH. Dry heating of NaH_2_PO_3_ solids derived from aqueous solutions (50 mM, at pH’s between 3.0–5.8) consistently afford PPi(III) in up to 30% after 6 days at 85 °C in the presence of a gas flow (either air or dinitrogen) or within a vacuum oven whereas starting from solutions at pH’s 6 and above show zero PPi(III) formation under the same conditions (Table 1 *upper*). Furthermore, dry heating Pi(III) solids from evaporated, simulated ocean water solutions [pH 3–4; 100 mM in Pi(III)] to 85 °C affords effective conversions to PPi(III) (40–60% after 24 h depending upon solution pH; Table 1 *lower*). Moreover, not only is initial solution pH a key factor influencing PPi(III) formation under these conditions, but mechanical action, presumably influencing particle size, and hence water-release, is also important. Thus, grinding dried samples of Pi(III) from distilled water prior to heating at 85 °C under gas flow results in up to 7 times the yield of PPi(III). Under the same conditions, dry heating solids from evaporated aqueous solutions of Pi(V) affords no PPi(V) formation.

We believe that geological scenarios for PPi(III) formation are prebiotically plausible; e.g., geothermal fields [23]. The PPi(III) precursor, phosphite, Pi(III), has been detected at 0.06 ± 0.02 μM concentrations in the geothermal Hot Creek Gorge system near Mammoth Lakes, California [24]. Furthermore, Pi(III) has been shown in laboratory tests to result from hydrothermal treatment of the P-mineral schreibersite, (Fe,Ni)_3_P [25,26,27,28,29], an important component of iron meteorites [30] and an identified product of lightning [31] and impact [32] induced strikes within phosphate-containing sites as well as being associated with (albeit recent) rock formations in Disko Island, Greenland [33].

**Figure 4:**
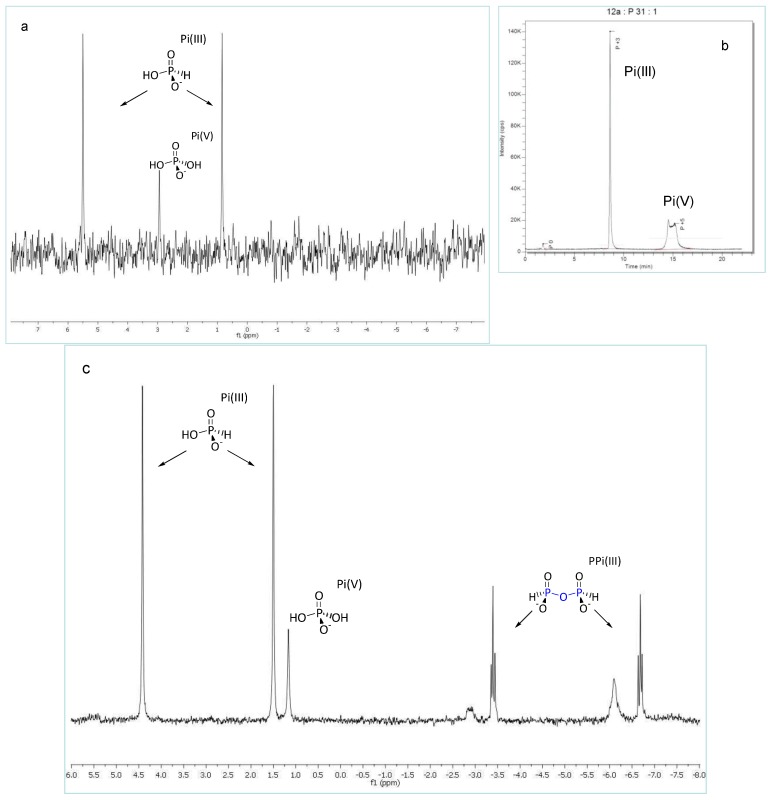
(**a**) ^31^P-NMR, sample of Hveradalur Lake incubated Fe_3_P sample A5 (Table 2), pre-heated (298 K, H_2_O solvent with D_2_O insert, 320 scans); (**b**) Speciation trace from HPLC-ICP-MS showing presence of both Pi(III) and Pi(V); (**c**) ^31^P-NMR of A5 post-heated (298 K, after heating to 90 °C for 72 h under flowing N_2_, H_2_O solvent with D_2_O insert, 2048 scans).

We report here *in situ* field hydrothermal studies on Fe_3_P, a schreibersite model, within a series of sub-glacial, low pH (1.6–4.7) and high temperature (40–94 °C) hydrothermal fluids in the Kverkfjöll volcanic mountain region of the Vatnajoküll glacier, Central Iceland. Post-incubation analyses viaICP-AES and ICP-MS identify solution P-levels between 0.5–22 mgL^−1^ (Table 2) and the principal P-product to be Pi(III) via ^31^P-NMR spectroscopy. Complementary results were obtained [34] by incubating samples of the type IIAB octahedrite iron meteorite Sikhote Alin, displaying exposed schreibersite inclusions, in the same geothermal field experiments (wherein are contained further details about the geological field site). Furthermore, the innovative combination of HPLC-ICP-MS analytical methods has allowed us to confirm Pi(III) presence via ion chromatography at low (sub-ppm) levels in all our *in situ* field Fe_3_P experiments (Table 2). Subsequent evaporation and dry-heating of these same geological fluid samples to 85 °C in the open air afforded PPi(III) as confirmed by ^31^P-NMR analysis of sample A5 (pre-heated A5 Figure 4a; post-heated A5 Figure 4c). Also displayed in Figure 4b is a speciation HPLC trace of pre-heated A5 which confirms the presence of both orthophosphate [Pi(V)] and H-phosphite [Pi(III)] in this sample.

### 3.2. Phosphonylation of Pi(V) with PPi(III) in Aqueous Solution. Formation of PPi(III–V) and PPi(V)

Identifying a viable chemical pathway from any primitive energy currency system to one based around PPi(V), can only strengthen the plausibility for the former and several such processes have been proposed [35,36,37,38,39,40,41,42,43]. We report such a pathway here employing the condensed P-oxyacid, PPi(III) which allows us to propose, for the first time, that coupling of a reduced oxidation state P-moiety to Pi(V) can activate the latter towards substrate level phosphorylation. Thus, we find that PPi(III) will phosphonylate orthophosphate, Pi(V), in aqueous solution at ambient temperature, achieving *ca*. 35% conversion to isohypophosphate [HP_2_O_6_^2−^; PPi(III–V)] after 16 h incubation at pH 7 (Figure 3). Moreover, the addition of MgCl_2_ or CaCl_2_ (50 mM) greatly enhances PPi(III–V) formation whereupon *ca*. 80% of total Pi(V) is converted to PPi(III–V) (Figure 4 and Table 3). Intriguingly, one can notice that not only does Ca^2+^ increase the rate at which PPi(III–V) is formed from PPi(III) and Pi(V), but also appears to accelerate the hydrolysis reaction of the former. This is potentially highly significant since it is points towards both rapid formation of and P-transfer from PPi(III–V), a chemical feature we are currently probing in more detail in this laboratory.

**Table 3:** Conversion (% of total P as determined by ^31^P-NMR, 298 K, H_2_O solvent with D_2_O insert, 320 scans) of an aqueous solution of PPi(III) (50 mM; 5 mL at pH 7, 298 K with additives of MgCl_2_ (50 mM) or CaCl_2_ (50 mM) in presence of Pi(V) (50 mM).

Time (h)	Control [% total P]		MgCl_2_ [% total P]		CaCl_2_ [% total P]	
	PPi(III)	PPi(III–V)	PPi(III)	PPi(III–V)	PPi(III)	PPi(III–V)
0	98.6	0	98.6	0	98.6	0
48	95.7	13.8	68.5	66.2	36.2	82.3
168	89.0	27.7	42.5	78.1	20.2	65.4
336	82.5	36.8	30.3	81.8	18.4	45.9
504	78.2	41.6	23.4	81.1	5.6	23.9

**Figure 5:**
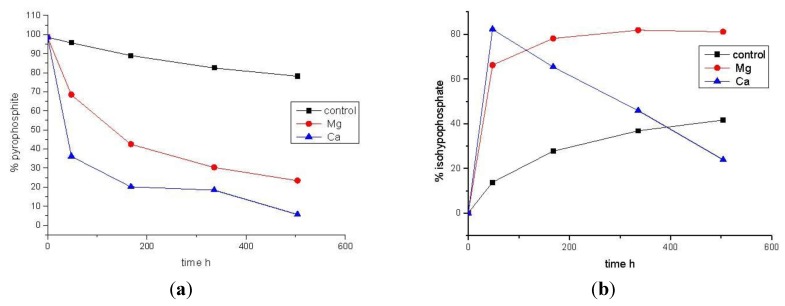
Graphical representations of the data shown in Table 3 for (**a**) PPi(III) consumption; and (**b)** PPi(III–V) production, from aqueous solutions of PPi(III) and Pi(V) (both 50 mM, pH 7, 298 K) clearly revealing the acceleratory effect of both PPi(III–V) formation and, for Ca^2+^ especially, a clear acceleration of PPi(III–V) hydrolysis.

It appears that the process of phosphonylating Pi(V) to PPi(III–V) *activates* the Pi(V) moeity. Thus, to a solution comprising PPi(III–V) 43.5%, Pi(V) 6.9% and Pi(III) 49.5% (0.1 M total P) was added Na_2_HPO_4_ and MgCl_2_·6H_2_O to achieve a solution with 2 equivalents of Pi(V) and 3 equivalents of Mg^2+^ with respect to total P. After heating this mixture to 80 °C in *aqueous solution* for 42 h, PPi(V) is detected at 1.7% of total P (by ^31^P-NMR; Figure 5), which translates to an *ca*. 7% conversion of the PPi(III–V) to PPi(V) under these mild conditions. Such coupling of Pi(V) moieties does not occur under such mild conditions in the absence of PPi(III) or alternative coupling agents [35,36,37,38,39,40,41,42,43].

## 4. Conclusions

In summary, we have demonstrated here that low pH geothermal fluids, such as those found within sub-glacial volcanic environments, are capable of producing reduced oxidation state P-compounds from schreibersite models (Fe_3_P) via hydrothermal processes. Furthermore, we have demonstrated that dry-heating these same field-site samples to 90 °C for three days affords the condensed P-species, pyrophosphite [PPi(III)], reactions that can be reproduced in both field and laboratory settings. Moreover, we find that PPi(III) is an excellent P-transfer reagent, reacting smoothly with orthophosphate [Pi(V)] in aqueous solution (pH 7; 298 K) to afford isohypophosphate, PPi(III–V). In turn, heating solutions of PPi(III–V) with further Pi(V) to 80 °C in aqueous solution, results in exchange of Pi(III) for Pi(V) and formation of pyrophosphate, PPi(V) in overall 7% conversion efficiency from PPi(III–V). This, we contend, provides evidence of the ability of the Pi(V) centre in PPi(III–V) being activated towards substrate-level phosphorylation to afford PPi(V) under relatively mild conditions (80 °C in aqueous solution) given that PPi(V) formation from Pi(V) does not occur under the same conditions in the absence of added PPi(III). Further studies are continuing to probe chemistries of PPi(III) and PPi(III–V) within putative Hadean geological environments.
